# Photoionization Dynamics of the Tetraoxo Complexes OsO_4_ and RuO_4_

**DOI:** 10.1021/acs.inorgchem.0c00683

**Published:** 2020-04-28

**Authors:** Luca Schio, Michele Alagia, Daniele Toffoli, Piero Decleva, Robert Richter, Oliver Schalk, Richard D. Thomas, Melanie Mucke, Federico Salvador, Paolo Bertoch, Davide Benedetti, Carlo Dri, Giuseppe Cautero, Rudi Sergo, Luigi Stebel, Davide Vivoda, Stefano Stranges

**Affiliations:** ‡ SBAI Department, Sapienza University, P.le A. Moro 5, I-00185 Rome, Italy; ¶ IOM-CNR Tasc, SS-14, Km 163.5, Area Science Park, Basovizza, I-34149 Trieste, Italy; § Dipartimento di Scienze Chimiche e Farmaceutiche, Università degli Studi di Trieste, Via L. Giorgieri 1, I-34127 Trieste, Italy; ∥ Elettra Sincrotrone Trieste, SS-14, Km 163.5, Area Science Park, Basovizza, I-34149 Trieste, Italy; ⊥ Department of Chemistry, University of Copenhagen, Universitetsparken 5, DK-2100 Copenhagen, Denmark; # Department of Physics, Stockholm University, Roslagstullsbacken 21, 10691 Stockholm, Sweden; ○ Department of Physics and Astronomy, University of Uppsala, Box 516, SE-75120 Uppsala, Sweden; ∇ Department of Chemistry and Drug Technologies, Sapienza University, P.le A. Moro 5, I-00185 Rome, Italy

## Abstract

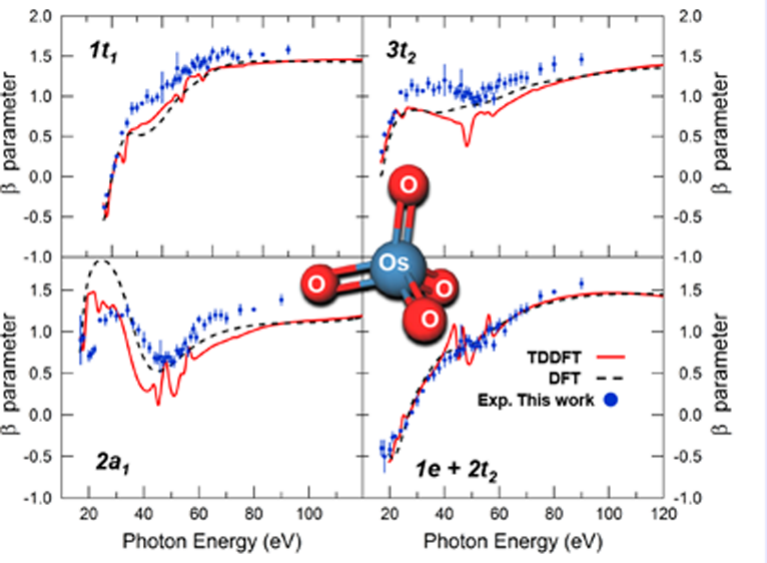

The photoionization dynamics of OsO_4_ and RuO_4_, chosen as model systems of small-size mononuclear heavy-metal complexes, has been theoretically studied by the time-dependent density functional theory (TDDFT). Accurate experimental measurements of photoionization dynamics as a benchmarking test for the theory are reported for the photoelectron asymmetry parameters of outer valence ionizations of OsO_4_, measured in the 17–90 eV photon energy range. The theoretical results are in good agreement with the available experimental data. The observed dynamical behavior of partial cross sections and asymmetry parameters has been related to both the coupling to the continuum of discrete excited states, giving strong modulations in the photon energy dependency, and the atomic composition of the initial ionized states, which determines the rate of decay of ionization probability for increasing excitation energies. Overall, an extensive analysis of the photoionization dynamics for valence and core orbitals is presented, showing good agreement with all the available experimental data. This provides confidence for the validity of the TDDFT approach in describing photoionization of heavy transition element compounds, with the perspective of being used for larger systems. Further experimental work is suggested for RuO_4_ to gather evidence of the sensitivity of the theoretical method to the nature of the metal atom.

## Abstract 2

In this work, the time-dependent density functional theory is used to calculate the photoionization dynamics of the valence and core ionizations of OsO_4_ and RuO_4_ complexes, which are chosen as model systems of small-size mononuclear heavy-metal complexes. Accurate experimental measurements of the branching ratios and photoelectron asymmetry parameters of outer valence ionizations of OsO_4_ provide a sound benchmark of the computational methodology.

## 1 Introduction

Mononuclear and polynuclear organometallic clusters are a class of interesting and technologically relevant complexes due to their widespread use in catalysis.^1^ From a fundamental point of view, the elucidation of the nature of metal–metal bonding in polynuclear complexes is of interest both for the rationalization of the relative stability and reactivity of these complexes and for the understanding of their ionization and fragmentation processes.^2^ The study of these metal clusters poses several challenges due to their complex electronic structure, bearing the signature of strong correlation effects and the need of relativistic corrections for the heavy metal centers,^3^ as well as their nontrivial nuclear dynamics which leads to a high degree of fluxionality^4,5^ and complex fragmentation dynamics.^2,6,7^

One of the principal tools for the investigation of molecular electronic structure is photoelectron spectroscopy (PE), which provides both ionization energies (IEs) and dynamical observables such as partial ionization cross sections and asymmetry parameters.^8−11^ While partial cross-section data, or branching ratios, have been widely reported, the complementary information provided by the asymmetry parameter β has scarcely been investigated for organometallic systems.

The present work has been motivated by the results of preliminary calculations of photoionization parameters of polynuclear heavy metal complexes, such as Ru_3_(CO)_12_ and Os_3_(CO)_12_, which are currently performed by our group with the aim to elucidate the correlation between the electronic structure and the photoionization observables. This task proves quite challenging due to the complex electronic structure of these systems,^4,5^ which makes even the assignment of the experimental spectra difficult. Computational methods to describe photoionization observables are well established for relatively simple and medium-sized organic molecules.^12−15^ To benchmark and assess the quality of the theoretical approach in the case of heavy metal atom containing systems, we have here investigated the tetraoxo complexes OsO_4_ and RuO_4_ as ideal model cases, since they are relatively rigid and small-sized systems for which the assignment of the valence photoelectron spectrum has been elucidated in detail on a firm basis.^16−19^

Most of the electronic structure calculations of these molecules published so far employed fairly simple computational approaches, such as the Xα method (in both nonrelativistic^20^ and quasi-relativistic^21^ versions), the Hartree–Fock–Slater (HFS) method,^22^ or the extended-Hückel method.^23^ More sophisticated nonrelativistic (NR) approaches, such as 2ph-TDA and ΔSCF-CI, were instead used by Green et al.^16^ More recently, theoretical studies of the valence photoelectron spectrum of OsO_4_^17−19^ confirmed the previously established spectral assignment of the PE bands. However, only one experimental study focused on the valence photoionization dynamics of OsO_4_,^16^ where relative partial photoionization cross sections have been obtained for the five outer valence PE bands. We are not aware of any experimental measurement of photoelectron angular distributions, and only one theoretical investigation of the OsO_4_ photoelectron dynamics exists,^19^ which is based on a plane waves approach and could not be validated for the β predictions by any experimental evidence.

This work fills a gap in the literature by providing theoretical partial photoionization cross sections and angular asymmetry parameters, over a wide photon energy range, of the first five valence ionization processes, as well as of selected core ionizations, using a state-of-the-art approach. Furthermore, the first experimental β values, measured for the outer valence ionizations of OsO_4_ in a wide energy range, are reported, thus providing detailed experimental information on the β dynamical behavior.

The paper is organized as follows. In section 2 we provide the relevant experimental and computational details. Results and discussions are presented in section 3, while conclusions and perspectives are summarized in section 4.

## 2 Methods

### 2.1 Experimental Section

The measurements were performed at the Gas Phase Photoemission beamline^24^ of the Elettra synchrotron radiation source (Trieste, Italy). Valence photoelectron (PE) spectra of the OsO_4_ molecule were recorded at fixed photon energies spanning the 17–90 eV range using the ARPES-TPES end station, equipped with a specifically designed rotatable hemispherical analyzer to detect photoelectrons (ARPES) and threshold photoelectrons (TPES) emitted by highly reactive and chemically aggressive gaseous species.^25−30^ The analyzer was operated in constant pass energy mode selecting 10 and 15 eV pass energies for PE spectra recorded below and above 22 eV, respectively. The molecular vapor generated by solid OsO_4_ (Sigma-Aldrich, purity ≥99%) at constant room temperature (24 °C) was admitted in the interaction region through a nonmagnetic hypodermic needle. A newly developed 2D position sensitive electron detector, successfully operated and used in the present experiment, largely improved the detection efficiency and resolution and allowed the use of a significantly lower target density, which was highly desired because of the known chemically aggressive nature of OsO_4_. The pressure in the ionization region could be kept below 1.0 × 10^–6^ mbar. A description of the detection technique and the snapshot spectral reconstruction and other details are given in the Supporting Information (SI).

The PE spectra were measured as a function of photon energy at two different detection angles with respect to the polarization axis of the linearly polarized radiation, namely at θ = 0° and θ = 54.7°. The asymmetry parameter β(*h*ν) was derived according to the equation β(*h*ν) = *R* – 1, where *R* is the ratio between the peak areas of the selected PE band obtained at the two different angles, *R* = *I*(0°)/*I*(54.7°). For each PE spectrum a corresponding background spectrum was recorded and subtracted. The accuracy of the β(*h*ν) parameter measurements was checked by measuring well-known asymmetry parameters of Ar and He, as references. The spectrometer resolutions used at 10 and 15 eV pass energy were 45 and 65 meV (fwhm), respectively, as measured by recording the Ar (3p)^−1^ doublet. All PE spectra were energy calibrated against the known IE values of N_2_ and Ar.

### 2.2 Theoretical Method and Computational Details

In the simpler mean field description of the scattering dynamics, a Kohn–Sham (KS) approach is employed whereby the ionized electron is scattered by a molecular potential uniquely described by the ground-state electron density. It is composed of three terms: the potential of the nuclear framework, the Hartree potential, and the exchange-correlation potential.^31^ Partial cross sections and asymmetry parameter profiles for each orbital ionization can be obtained through standard formulas^32^ involving phase shifts and transition dipole matrix elements
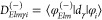
1where γ denotes a component of the dipole operator, *d*, φ_*i*_ is any occupied MO in the ground-state KS Slater determinant, while φ_*Elm*_^(−)^ is a properly normalized eigenvector of the KS Hamiltonian for a given energy *E* in the continuum spectrum.^12^ In our implementation we employ a discretization of both bound and continuum wave functions in a multicentric basis set of B-spline functions.^33^

The KS method is unable to describe important resonant phenomena such as autoionization, which usually affect the ionization dynamics of transition metal compounds (e.g., Super Coster–Kronig (C–K) decays). Within the linear-response TDDFT approach, the central variable becomes the effective time-dependent KS potential, *v*^*SCF*^, which (in the frequency domain) can be expressed as
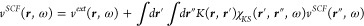
2where we have invoked the adiabatic local density approximation for the kernel *K*,^34^ and χ_*KS*_ is the KS susceptibility, while *v*^*ext*^ is the external dipole potential.

When both *K* and χ_*KS*_ kernels are represented in the LCAO basis, the expansion coefficients of the *v*^*SCF*^ potential are obtained as the solution of the linear system
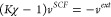
3and TDDFT transition matrix elements entering in the computation of the photoionization observables are obtained by replacing the appropriate component of *v*^*SCF*^ in place of the dipole operator in eq 1.^12,15,35^

Both RuO_4_ and OsO_4_ have a *T*_*d*_ equilibrium geometry, with an M–O bond length of 1.706 and 1.711 Å for M = Ru, Os, respectively.^36^ For both molecules, the ground-state electron density has been obtained by using the ADF quantum chemistry code.^37,38^ Scalar relativistic effects have been included in the framework of the zero-order regular approximation (ZORA^39,40^), while a TZP basis set optimized for ZORA calculations and taken from the ADF database has been used for all atoms. The self-consistent electron densities are then projected on the LCAO B-spline basis set and used to define the Kohn–Sham Hamiltonian for the subsequent scattering calculations. The full molecular point-group symmetry is employed in all calculations. The B-splines of the OCE expansion, centered on the metal atom, are defined in a radial grid of knots extending up to 20.0 au and with a linear step size of 0.2 au, while the radial grid on the O atoms extends up to 1.1 au The truncation of the OCE expansion has been fixed at *l*_*max*_^*OCE*^ = 20, while *l*_*max*_ = 2 for the off-center spheres. In the TDDFT calculations we included the coupling among all main-line dipole channels originating from the outer valence and O 2*s* ionizations, together with selected core levels of the metal atom; these were the 5*s*, 4*f*, and 5*p* for Os and 4*s* and 4*p* for Ru. For an easier comparison with the experimental data, strong and sharp features in the computed TDDFT partial cross sections and asymmetry parameter profiles, due to autoionization resonances, are smoothed by convolution with Gaussian functions with full width at half-maximum of 1.0 eV. Ionization potential energies have been obtained at the KS ΔSCF level by employing the BP86 *xc* potential^41,42^ and a TZP basis set, while relativistic corrections were included within the ZORA formalism.

## 3 Results and Discussion

In the following sections we will consider the valence ionization dynamics of the two tetraoxo complexes. For OsO_4_, we compare KS/TDDFT results with experimental cross-section data taken from refs (16 and 43), as well as newly acquired asymmetry parameter profiles from this work. We will also address whether purely atomic effects, such as the presence of a Cooper minimum in partial cross sections from orbitals with metal *d* character, carry over in a molecular environment. Cross-section data and asymmetry parameters for selected inner-valence and core ionizations of OsO_4_ and RuO_4_ are instead discussed in a separate section of the SI.

### 3.1 Valence Ionization Energetics

Earlier measurements of the He–I PE spectra of both tetraoxo complexes were reported by Diemann et al.^44^ and Burroughs et al.,^45^ while a later work by Green et al.,^16^ using synchrotron radiation, was instrumental in resolving long-standing controversies^43^ concerning the energy ordering of the outer valence molecular orbitals (MOs) and the assignment of the outermost bands of the PE spectrum of OsO_4_ and RuO_4_.

Both *d*^0^ MO_4_ species have a valence electronic structure of the type
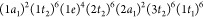
where the traditionally used numbering scheme of the valence MOs^16,45^ has been adopted.

**Figure 1:**
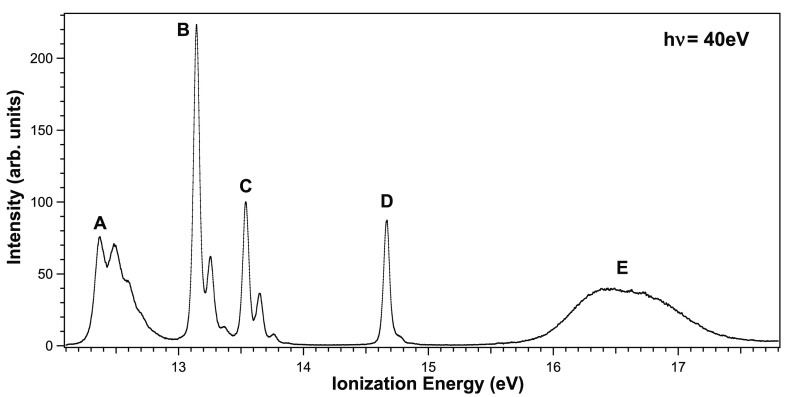
Outer-valence PE spectrum of OsO_4_ recorded at *h*ν = 40 eV and θ = 54.7° (magic angle), using 15 eV pass energy.

The valence PE spectrum of OsO_4_ recorded in this work at *h*ν = 40 eV and θ = 54.7°, the “magic angle”, is shown in Figure 1. The five bands, denoted as features A–E in the figure, are in excellent agreement with the published PE spectra of OsO_4_, and all but band E exhibit vibrational structure. This vibrational structure has been suggested to be mainly due to the ν_1_(a_1_) “breathing” mode excitation,^45^ although in band A the contribution from bending mode excitations of *e* symmetry has also been pointed out.^19^

The assignment of the five bands of OsO_4_ (see Table 1) to specific orbital ionizations in order of increasing IE is described as follows.^17−19,46^ The first band, A, is assigned to the ^2^T_1_ ionic state corresponding to the ionization from the 1t_1_ MOs. The next two bands, B and C, separated by 0.4 eV in OsO_4_ and by only 0.09 eV in RuO_4_ (see Table S1 of the SI), are assigned to the spin–orbit components^21^ associated with ionization from the 3t_2_ MOs with strong contribution of O 2*p* atomic orbitals (AOs), namely the ^2^T_2_ ion state. The fourth band, D, is assigned to the ^2^A_1_ ion state, while the fifth band, E, encompasses the remaining outer valence MO ionizations (^2^E and ^2^T_2_ ion states). The valence MOs 1a_1_ and 1t_2_ are essentially symmetry adapted linear combinations of O 2s AOs, whose ionizations are energetically not accessed by He–I radiation. This assignment then carries over to RuO_4_.

**Table 1:** Comparison between ZORA LB94/TZP MO Energies, ΔSCF IEs from This Work, and Theoretical (TS,^46^ EOM-SOCCSD,^18^ and CASSCF^19^) and Experimental IEs for OsO_4_^f^

band	ion state (MO)	ΔSCF^a^	–ε_*KS*_^*LB*94^^a^	TS^b^	EOM-SOCCSD^c^	CASSCF^d^	exp^e^	exp^a^	AO character^a^
A	4U′ (1t_1_)	12.61	15.14	13.315	12.41	11.33	12.35	12.37	O_2*p*_
A	2E′ (1t_1_)	12.78			12.47	11.33			
B	3U′ (3t_2_)	13.26	15.84	13.684	13.23	12.02	13.14	13.14	96% O_2*p*_/2% Os_6*p*_
C	2E″ (3t_2_)	13.62			13.50	12.32	13.54	13.54	
D	1E′ (2a_1_)	14.97	17.26	14.986	14.86	13.62	14.66	14.67	85% O_2*p*_/7% O_2*s*_/8% Os_*ns*_ (*n* = 6,7,8)
E	1E″ (2t_2_)	16.78	19.26	17.823	17.30	15.28, 15.31	16.4–16.8	16.33–16.78	47% O_2*p*_/9% O_2*s*_/39% Os_5*d*_
E	2U′ (2t_2_)	16.89			17.35	15.7	16.4–16.8	16.33–16.78	
E	1U′ (1e)	17.35	19.42	18.512	17.92	15.78, 15.81	16.4–16.8	16.33–16.78	56% Os_5*d*_/44% O_2*p*_

a From this work.

b From ref (46).

c From ref (18).

d From ref (19).

e From ref (45).

f This work and from refs (45 and 46). All energies are in eV. AO contributions to the MOs calculated at the LB94/TZP level are also reported.

In Table 1 we report a comparison between experimental and calculated IEs for the first five PE bands of OsO_4_, while the corresponding data for RuO_4_ are reported in Table S1 of the SI.

From the IE values reported in Tables 1 and Table S1, we observe that the ΔSCF KS procedure provides good estimates of the valence IEs, especially in the case of OsO_4_, since discrepancies with the available experimental data are of the order of 0.2 eV. The KS ΔSCF estimated spin–orbit splitting of the ^2^T_2_ ionic state (bands B and C in Figure 1), which is 0.36 eV for OsO_4_ and 0.11 eV for RuO_4_, is in good agreement with the corresponding experimental observations, i.e., 0.40 and 0.09 eV, respectively, indicating that spin–orbit effects are rather accurately described at the ZORA-KS level. The accuracy of the ΔSCF estimates given in this work is, on average, higher than that of both the TS values reported in ref (46) and those reported in ref (16) obtained by more sophisticated ab initio methods and comparable to the best ab initio values.^18^ Regarding the AO composition of the ionized orbitals^16,19,21^ (see Table 1 and Table S1 of the SI), for both complexes the 1t_1_ MOs are nonbonding, 3t_2_ and 2a_1_ have dominant contributions from the O 2*p* AOs, while the metal *d* orbitals are heavily hybridized with ligand O 2*p* AOs in the two innermost MOs 2t_2_ and 1e of the valence shell, which have a strong bonding character. This last character has strong implications on the substantial difference of the cross-section behavior for ionization from these MOs from the pure atomic behavior characteristic of ionizations from atomic Os 5*d* and Ru 4*d* orbitals.

**Figure 2:**
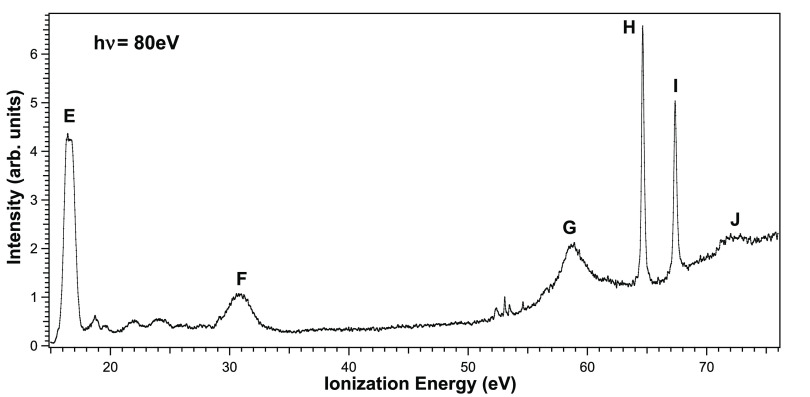
High-energy valence PE spectrum of OsO_4_ recorded at *h*ν = 80 eV and θ = 54.7° (magic angle), using 15 eV pass energy. The very weak spectral contaminations at 52–55 eV IEs are due to the outer valence ionizations from 40 eV photons emitted by the bending magnet undulator section and passing through the monochromator as first-order diffraction light, while the spectrum is obtained from 80 eV photons, undulator first harmonic emission, selected by the monochromator as second-order diffraction light.

In Figure 1 the unusual width and shape of band E in the region 16.33–16.78 eV should be noted. Although comprising ionizations from two groups of MOs (three ionic states when relativistic effects are included), the band broadening might largely originate from vibronic excitations, because of the bonding character of the ionized orbitals. A main contribution to the band broadening can also be due to a strong breakdown of the one-particle approximation, with a dense manifold of satellite states, which was predicted in ref (17), where, however, final state configuration interaction effects for the outer ionizations seem to be significantly overestimated, as this spectral region is not accurately described. The importance of the electron correlation in the final state has also been pointed out for band E by the authors of a recent paper.^19^ Nevertheless, a clear presence of satellites is seen in our experimental spectrum at higher energies, namely in the 19–30 eV IE region (see Figure 2). A prominent peak, band F in Figure 2, centered at 30.85 eV, is clearly assigned mainly to O 2s ionizations, possibly broken by many-body effects. Two sharp peaks due to Os 4*f*_7/2_ and 4*f*_5/2_ ionizations, located at 64.65 and 67.37 eV (features H and I), are between the broad Os 5*p*_3/2_ and 5*p*_1/2_ peaks (G and J), observed at 58.80 and 71.98 eV, respectively, which show a very large spin–orbit splitting, and a large lifetime broadening due to Coster–Kronig decay.

### 3.2 Valence Ionization Dynamics

**Figure 3:**
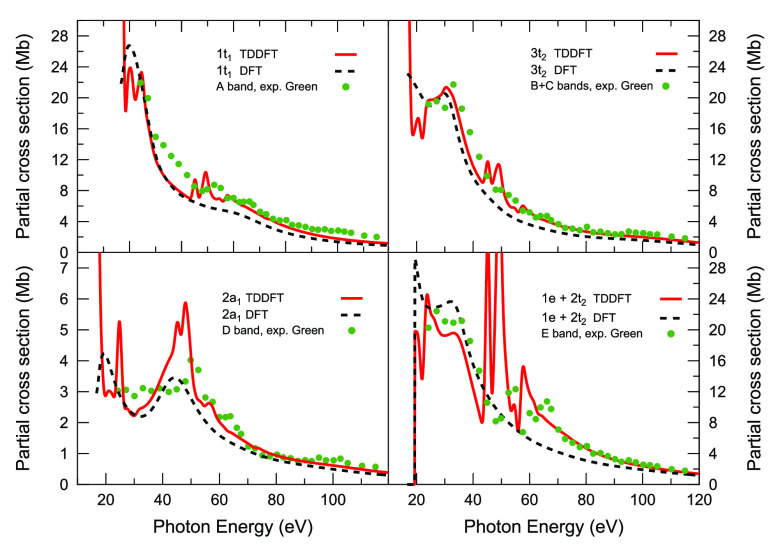
KS (black broken line) and TDDFT (red solid line) partial cross sections for the outer valence ionizations of OsO_4_. Also shown are the experimental data taken from ref (16) (green circles).

**Figure 4:**
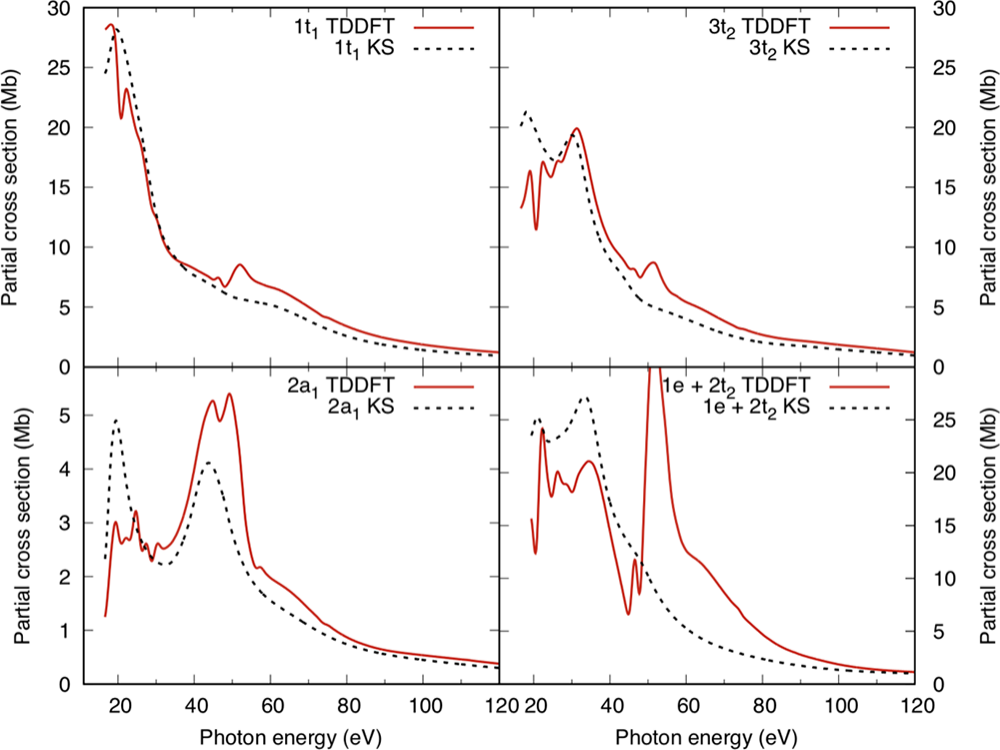
KS (black broken line) and TDDFT (red solid line) partial cross sections for the outer valence ionizations of RuO_4_.

Partial cross sections for the ionization of the outer-valence orbitals of OsO_4_ and RuO_4_ are reported in Figure 3 and Figure 4, respectively. The experimental cross-section data of ref (16) for OsO_4_ are also shown for comparison in Figure 3. From the intensity profiles for the OsO_4_ complex (Figure 3), we can conclude that there is a general good agreement with the available experimental data. Apart from the description of the autoionization resonances, which is given only by the TDDFT method, whose predicted magnitudes appear strongly damped in the experiment, the most clear case where TDDFT is needed for a quantitative agreement with the experimental cross sections is for the ionizations of the *d* metal-based MOs 1e and 2t_2_ in the 50–100 eV photon energy range (lower right panel in Figure 3), where the KS curve substantially underestimates the experimental intensity.

Although the super Coster–Kronig decay of the 5*p* → 5*d* singly excited state on the available open continua can be inferred from the scatter of the experimental points around 50–60 eV, the cross-section modulation is much more pronounced in the TDDFT profiles. Moreover, the “double hump”, visible in the experimental cross-section curve of band E (ionization of the 1e + 2t_2_ orbitals) with an energy separation of 12 eV that correlates with the spin–orbit splitting of the two ^2^P Os 5*p*^–1^ subshells, cannot be reproduced by our nonrelativistic TDDFT method. This latter, however, can reproduce, although hidden by the convolution procedure, the first few members of the 5*p* → *nd* Rydberg series converging to the Os 5*p* threshold. These features are not resolved in the experimental data likely because of their low cross sections and short excited-state lifetimes.

Compared to the other outer valence ion states, the partial cross section for the ionization of the 2a_1_ state shows a distinctly different behavior, as for the magnitude, as well as for the presence of a maximum at around 45 eV in the KS profile. This latter feature is also predicted at the TDDFT level, although here it is superimposed on the series of autoionizing 5*p* → *nd* excitations. We ascribe the resonant enhancement in the KS curve around 45 eV to the occurrence of a shape resonance in the t_2_ continua, where major contributions to the partial cross section are given by *l* = 4 and *l* = 7 partial waves. This effect does not affect significantly the other calculated partial cross sections, as confirmed by the experimental observations.^9,16^

A comparison between Figure 3 and Figure 4, which shows the KS and TDDFT partial cross sections for the valence ionizations of RuO_4_, suggests that the strict similarity observed between the corresponding data in nonresonant regions, and which has been discussed above for OsO_4_, reflects the similar composition of MOs in terms of constituting AOs of the valence orbitals of the two tetraoxo complexes. Also for RuO_4_ the cross-section profile for the 2a_1_ ionization is lower in magnitude than that of the other valence ionizations, and it is characterized by a resonant enhancement at around 45 eV photon energy. Perhaps the most noticeable difference between the two complexes shows up in an increased probability for the decay of the 4*p* → 4*d* excited state in the 1e + 2t_2_ continua in the case of RuO_4_. This behavior has recently been observed for the polynuclear metal complexes Os_3_(CO)_12_ and Ru_3_(CO)_12_^2^ and could be also present in the case of OsO_4_ and RuO_4_. Similar results between second and third row transition metal compounds have also been reported for the Group 6 hexacarbonyls, Mo(CO)_6_ and W(CO)_6_,^47^ and the Group 8 metallocenes, Ru(η-C_5_H_5_)_2_ and Os(η-C_5_H_5_)_2_.^8^ This could be eventually verified for the tetraoxo complexes by further experimental investigation of RuO_4_.

**Figure 5:**
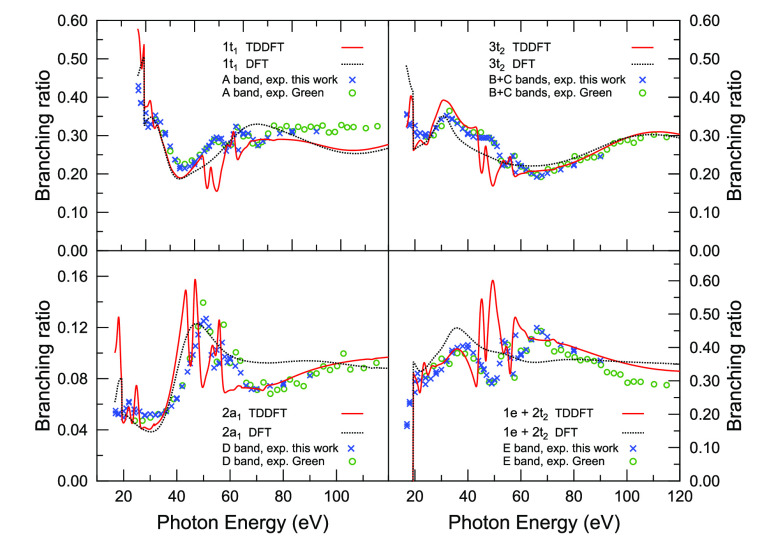
Experimental and theoretical photoionization cross-section branching ratios for the outer valence ionization of OsO_4_ (bands A–E) as a function of photon energy. The two sets of experimental data are from this work (blue cross marks) and are derived from ref (16) (green open circles). The theoretical data are obtained in this work and calculated at different levels of theory, KS (black dotted line) and TDDFT (red solid line).

The experimental data obtained in this work allowed the measurement of the photoionization cross-section branching ratios for the first five PE bands of OsO_4_ as a function of photon energy. This data set is presented in Figure 5 together with the same experimental branching ratios derived from the relative cross sections measured in ref (16). There is an excellent agreement between the two independent experimental data sets, whose merging over a wide photon energy range provides accurate information on the dynamical behavior of the partial cross sections. Also included in Figure 5 are the corresponding branching ratios calculated at KS and TDDFT levels of theory from this work. The calculated and experimental results are in excellent quantitative agreement. However, structures in the autoionization regions are slightly overestimated in the TDDFT results, and some consistent energy shift is apparent.

**Figure 6:**
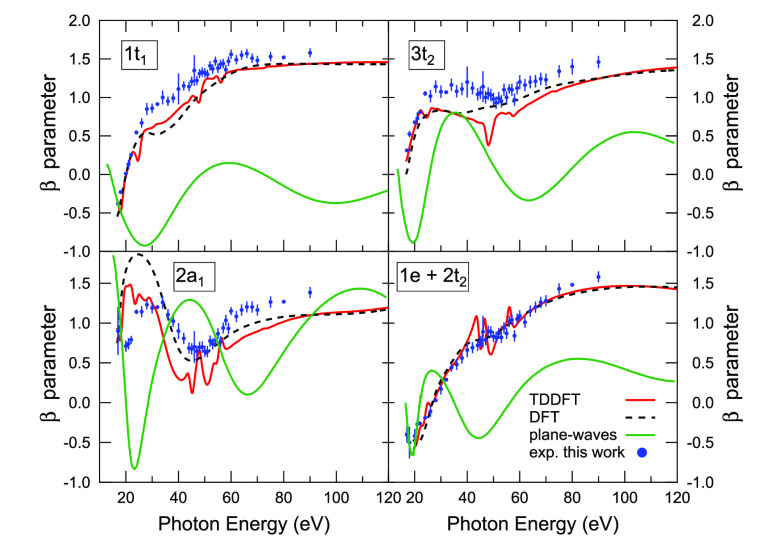
Asymmetry parameter profiles for the outer valence ionizations of OsO_4_ calculated by KS (broken line) and TDDFT (solid red line), as well as obtained experimentally (blue circles). β profiles calculated by the plane waves based method in ref (19) are also reported for a comparison (solid green line).

Turning to the angular distribution of photoelectrons, the asymmetry parameters for the outer valence ionizations of OsO_4_ calculated by KS and TDDFT are presented in Figure 6. It is interesting that a general good agreement between the KS and TDDFT estimates is observed over the whole investigated energy range, with the exception of the narrow energy windows corresponding to resonant regions. This applies to all ionizations with the only notable exception being that from the 2a_1_ MO, where the oscillation in the asymmetry parameter profile is somewhat more pronounced at the KS level. In all other cases, the curves are characterized by a rise from negative values close to the thresholds to positive values. The high-energy behavior of the β profiles is MO dependent: very flat for the 1t_1_ ionization, with a broad maximum in the case of the 3t_2_ and for the ionization from the 1e and 2t_2_ MOs, while damped oscillations are superimposed on a rising background for the 2a_1_ ionization.

The experimental β parameters for the outer valence ionizations of OsO_4_ obtained in this work are shown in Figure 6 together with the calculated theoretical values. The agreement between the KS/TDDFT estimates and the experimental data is overall very satisfactory for all PE bands. It can be considered quantitative for the E band (1e^–1^ + 2t_2_^–1^ ionizations), while the theoretical predictions underestimate the experimental β values for the 1t_1_^–1^ and 3t_2_^–1^ ionization, although the shape of the experimental profiles is closely reproduced by the calculated curves. The agreement between theory and experiment seems to deteriorate for the 2a_1_^–1^ ionization in the low energy region. Here the experimental data suggests a more damped β oscillation in the 40–60 eV energy range of the shape resonance, in somewhat closer agreement with the TDDFT predictions. Above 60 eV photon energy, the experimental β values are instead consistently underestimated by both KS and TDDFT. The valence photoionization dynamics of OsO_4_ has recently been theoretically investigated by calculating cross sections and β parameters as a function of photon energy.^19^ The β profiles, calculated by a plane waves based method by these authors, are also reported in Figure 6 for a comparison with the experimental and theoretical data obtained in this work. The accurate experimental β values clearly demonstrate that the plane waves method provides a poor description of the photoionization dynamics of OsO_4_, and this is also observed in comparing cross-section profiles with the experimental curves.^19^

Asymmetry parameter profiles have also been calculated at KS and TDDFT levels for the valence ionizations of RuO_4_ (see the SI) and displayed a behavior very similar to those exhibited by the corresponding OsO_4_ curves.

To conclude the discussion on the valence ionization of the two tetraoxo complexes, it is interesting to inquire whether the Cooper minima, whose presence in the ionization from atomic Os 5*d* and Ru 4*d* AOs has been calculated at 190 and 100 eV, respectively, employing a similar KS approach, based on the X_α_ exchange-correlation potential,^48^ do still affect ionizations from MOs that are involved in the formation of the covalent M–O bonds and have substantial metal *d* orbital character. A Cooper minimum is a purely atomic effect: it occurs in the ionization from AOs with radial nodes, and its energy position generally increases with increasing atomic number.^49,50^ The TDDFT partial cross section for the (1e)^−1^ and (2t_2_)^−1^ ionizations together with their sum is reported in the upper and lower panels of Figure S6 of the SI for OsO_4_ and RuO_4_, respectively, in the energy region where the Cooper minima in the atomic cross sections have been predicted.^48^ In the same figure the calculated Os 5*d* and Ru 4*d* cross sections are also reported. Due to the presence of such minimum in the atomic *d* orbital ionizations, the cross section of this AO exhibits in the low energy region a steeper decrease than that shown by AOs without radial nodes, e.g., the O 2*p* orbitals. The computed cross section of atomic *d* orbitals shows, in the low energy region, a decrease larger than that of TDDFT cross sections of MOs with pronounced *d* metal character, namely 1e and 2t_2_ in Figure S6, this being due to the significant O 2*p* AO’s contributions to the MO character, as reported in Table 1 and Table S1 for OsO_4_ and RuO_4_. This interpretation is based on the well-known Gelius intensity model.^51^ The cross section for the 1e orbital ionizations is characterized, in both OsO_4_ and RuO_4_, by a somewhat larger decrease than that corresponding to the 2t_2_ MOs. This is due to the larger metal 5*d* (4*d*) contribution to the 1e MOs. The absence of a clear minimum in the TDDFT cross sections is, however, a clear indication of the metal–oxygen mixed character of the 1e and 2t_2_ MOs due to the covalent M–O bonds formation. The presence of a Cooper minimum was also reported in the first two PE bands of Os(CO)_5_ by Hu et al.,^52^ although it was difficult to distinguish this feature from molecular effects induced by the CO ligands. The electronic structure of Os(CO)_5_ is however different from that of OsO_4_, since the lower ionic nature of the metal–ligand bond in the former reflects a larger mixing of Os 5d with O 2*p* AOs in the valence bonding MOs. Experimental data on the valence photoionization dynamics of RuO_4_, which are presently unavailable, would be highly desired since they allow for an extension of the above discussion on theoretical data to experimentally observed dynamical observables.

## 4 Conclusions

The photoionization dynamics of the tetraoxo complexes OsO_4_ and RuO_4_ has been calculated by the time-dependent density functional theory (TDDFT) that is implemented in a linear combination of atomic orbitals (LCAO) scattering code, which uses a basis set of B-spline functions.^12,15,53^ Accurate experimental asymmetry parameters and partial cross-section branching ratios of the outer valence ionizations of OsO_4_ have been measured as a function of photon energy by linearly polarized synchrotron radiation. The theoretical data have been compared with all the available experimental data, and a very good agreement has been found. Particularly, the new experimental β curves provided a benchmarking test to validate the accuracy of the TDDFT method in describing the photoionization dynamics of OsO_4_. Furthermore, the method has been clearly found to be much more accurate than a recent theoretical approach used for investigating the photoelectron dynamics of OsO_4_. The obtained results suggest that the TDDFT, as here implemented, can be used to accurately describe larger size molecular systems containing heavy atoms, for which an efficient method is not yet available.

Valence and core orbitals ionizations have been theoretically characterized for both tetraoxides OsO_4_ and RuO_4_. Overall, the photoionization dynamics of the two complexes displays signatures of both single particle and many-body effects. Purely single particle effects include the presence of shape resonances in selected ionization channels (2a_1_ orbital ionization) and the presence of Cooper minima in ionizations from MOs that retain an atomic character (Os 5p and Ru 4p AO-based). Many body effects include the super Coster–Kronig decay of *n*p → *n*d giant resonances that profoundly affect the ionization dynamics around 60 eV photon energy and whose effects are predicted to be stronger in RuO_4_ compared to OsO_4_.

The occurrence of a Cooper minimum in ionization from MOs with *d* metal character has been ruled out by our theoretical predictions, due to a strong hybridization of the *d* metal orbitals with ligand orbitals.

The present work should stimulate further experimental investigation on the photoionization dynamics of heavy transition metal compounds in different electronic situations and more complex systems. These data will allow for verification of the accuracy of the proposed methodology in a sufficiently large variety of molecular structures.

## Supporting Information Available

Description of experimental setup, experimental outer valence PE spectra of OsO_4_ used to derive β asymmetry parameter, selected KS/TDDFT results not included in main text (PDF)

## Dedication

This work is dedicated to the memory of our colleague and friend De Luisa Sandi, for his valuable contribution to the research activities of IOM-CNR Institute.
