# Supplementary material for: Photoionization Dynamics of the Tetraoxo Complexes OsO4 and RuO4
Source: Inorg Chem. 2020 Apr 28;59(10):7274–82. doi: 10.1021/acs.inorgchem.0c00683 (PMC8007099; doi:10.1021/acs.inorgchem.0c00683)
Supplement: Supplementary file 1 — ic0c00683_si_001.pdf [file ic0c00683_si_001.pdf]

Supporting Information for: Photoionization dynamics of the tetraoxo complexes OsO_4 and RuO_4

Luca Schio,[†,‡] Michele Alagia,[‡] Daniele Toffoli,[*,¶] Piero Decleva,[¶] Robert Richter,[§] Oliver Schalk,[∥] Richard D. Thomas,[⊥] Melanie Mucke,[#] Federico Salvador,[‡] Paolo Bertoch,[‡] Davide Benedetti,[‡] Carlo Dri,[‡] Giuseppe Cautero,[§] Rudi Sergo,[§] Luigi Stebel,[§] Davide Vivoda,[§] and Stefano Stranges[*,@,‡]

†*SBAI Department, Sapienza University, P.le A. Moro 5, I-00185, Rome, Italy.*

‡*IOM-CNR TASC, SS-14, Km 163.5, Area Science Park, Basovizza, I-34149, Trieste, Italy.*

¶*Dipartimento di Scienze Chimiche e Farmaceutiche, Università degli Studi di Trieste, Via L. Giorgieri 1, I-34127, Trieste, Italy.*

§*Elettra Sincrotrone Trieste, SS-14, Km 163.5, Area Science Park, Basovizza, I-34149, Trieste, Italy.*

∥*Department of Chemistry, University of Copenhagen, Universitetsparken 5, DK-2100 Copenhagen, Denmark.*

⊥*Department of Physics, Stockholm University, Roslagstullsbacken 21, 10691 Stockholm, Sweden.*

#*Department of Physics and Astronomy, University of Uppsala, Box 516, SE-75120, Uppsala, Sweden.*

@*Department of Chemistry and Drug Technologies, Sapienza University, P.le A. Moro 5, I-00185, Rome, Italy.*

E-mail: toffoli@units.it; stefano.stranges@uniroma1.it

1 The ARPES-TPES end station upgrade, new two-dimensional position sensitive electron detector

The experimental photoelectron spectra of the OsO_4 molecule were recorded using the ARPES-TPES end station, which has been operated at beamlines of the Elettra synchrotron radiation facility of Trieste (Italy). The apparatus, specifically designed to study highly reactive and chemically aggressive gaseous species, has already been described in detail elsewhere (see references cited in the main article), and so here we present a detailed description of the newly developed 2D position sensitive electron detector, which has been operated successfully for the first time in the present investigation.

The photoelectron spectrometer of the end station, shown in Figure S1, is a hemispherical analyzer with 180° sector and 100 mm mean radius that can rotate with high precision with respect to the polarization plane of the linearly polarized synchrotron radiation from an angle θ=0° to θ = 74°, thus allowing accurate photoelectron angular distribution measurements. Two new upgrades, an asymmetric fringing field corrector[1] and a 2D position sensitive electron detector, reported in the Figure, as Bias Electrode and 2D Detector Assembly, are mounted at the entrance and the exit of the hemispherical sector, respectively, and significantly improved the performance of the analyzer. The 2D position sensitive detector, which replaced the previous single channeltron device, is a fully home-made development[2] based on the crossed delay line technology.[3,4] The key parts of the detector are shown in Figure S2, which shows an exploded view of the assembly. We highlight the 40 mm diameter Chevron Multi-Channel-Plate (MCP) stack followed by a high voltage decoupling system, based on a resistive anode, and a Printed Circuit Board (PCB) two cross delay line system. The latter is based on the work by G. Cautero et al. and is described elsewhere.[5,6] The assembly is mounted on the exit plate of the hemispherical analyzer, as shown in Figure S1. The heart of the detector consists of two "microstrip" transmission lines in a meander shape. The position of the charge centroid from the MCPs is calculated by measuring the arrival times of the pulses, which propagate to the ends of the two delay lines (X and Y). The X and Y start and stop signals

coming from the detector are coupled out of the UHV section through four SMA connectors and immediately amplified by four properly customized preamplifiers, in this way reducing the pick-up noise. The signal from the detector is processed by a 4-channel time-to-digital-converter (THR02-TDC). In the present work, the THR02-TDC is configured for the 2D delay line detector such that the instrument can compute the (XY) position and arrival time with respect to an external/internal trigger signal, for each detected particle. The THR02-TDC has 4 independent input channels, with four separate time converters, coupled to four Constant Fraction Discriminator (CFDs). The start and stop ECL (emitter-coupled logic) signals produced by the CFDs are fed to the Time-to-Digital converter (TDC) devices integrated in the THR02-TDC. The CFDs receive an analog input which is converted by the discriminators to a digital pulse, preserving the temporal information; the task of the TDCs is to establish in which instant the event occurred and provide a digital representation of the event elapsed time.

The software of the previous single channeltron detection system has also been upgraded in order to efficiently record and process the imaging data produced by the 2D detector. A host client server protocol is adopted for the data transfer between the THR02-TDC and the acquisition software. The particles hit positions and arrival times recorded by the cross delay anode are processed by the THR02-TDC hardware and the host PC software, and are converted into a bi-dimensional image that represents the photoelectron spectrum projection on the analyzer's exit plane. The image is represented by the software as a matrix of 1000$\times$1000 time bins. The detector, constructed from a 30 mm$\times$30mm cross delay line and a set of three 29mm$\times$29mm masks, is placed between the MCPs and the resistive anode. This arrangement corresponds to an active surface fraction of 800$\times$800 time bins from the entire 1000$\times$1000 matrix. The bin digital resolution is $\sim$27 ps, while the temporal precision (FWHM), being equal to three bins, corresponds to a spatial dimention of 90-100 microns. One dimension of the 2D image is related to the electron kinetic energy dispersion, while the other dimension corresponds to the angular dispersion of the electron beam. Through a calibration procedure based on recording photoelectron spectra of different gases at different pass energies, the bin time scale is converted to the kinetic energy scale of the photo-

electron beam impinging on the detector. The image is processed by the acquisition software as follows. For each kinetic energy scanned by the analyzer, a single snapshot image, centered at the selected kinetic energy, is acquired and integrated in a 1D histogram by selecting a 700$\times$700 bin region of interest, in order to minimize the effect at the detector edges that can affect a uniform reconstruction of the spectrum. The temporal bin scale of the 1-D histogram is then converted to the corresponding kinetic energy scale depending on the energy dispersion at the selected pass energy. Finally, the histograms are summed according to their kinetic energy, and the intensity corresponding to each kinetic energy value is weighted by the number of times it has been recorded. The 2D image histograms are also saved in a matrix, and can be reanalyzed by other software. The raw images, the reconstructed photoelectron spectrum, and the matrix containing all the 1D-histograms are saved for each acquisition. It is possible to freely choose the binning of the images, as well as that of the reconstructed spectrum, independently from the kinetic energy step used in the scan. Although using a 2D position sensitive electron detector allows a significant improvement of the counting rate with respect to the single channeltron, it is worth mentioning that a non negligible fraction of particle hits is discarded, since only electron impact events generating all the four signals from the two delay lines, and which have a time difference in the calculated (X, Y) coordinates that falls in a time window between 3ns and 10 ns (limits which depend on the detector settings), are considered. However, the use of 2D cross delay line anode detectors exhibits several advantages with respect to a channeltron device. The high spatial and temporal resolution makes it simple to integrate counts over the desired distance along the non-dispersive direction. The direct observation of the projection of the electron beam image on the detector input surface at the exit plane of the analyzer is a useful tool to monitor the behaviour of the electron optics, in particular for optimizing the spectrometer performances. The most important feature of a 2D detector is the ability to acquire the entire spectrum in a single shot, by exploiting the energy dispersion of the analyzer. In the present case, typical kinetic energy windows spanned by the detector ranges from 0.7 eV, at a 5 eV pass energy, to approximately 2eV at 15eV pass energy. The acquisitions can take place either in single shot mode, exploiting the detector energy dispersion, or in scan mode, where

lenses and hemispheres voltages are changed in steps, extending the acquisition over the desired energy range. In the latter case, single-shot data are scaled and summed according to the voltage settings. The improvements in the detection sensitivity, acquisition time and resolution of such 2D detectors are of great importance in gas-phase experiments, particularly for very low density samples, where the counting rate can be of the order of 10-50 c/s, as is likely when studying highly reactive and chemically aggressive species (this work).

The fringing field effects, typical for the electron hemispherical analyzers, have been partially corrected by the use of a bias electrode positioned in an asymmetric configuration immediately after the entrance slit, and between this slit and the analyzer inner hemisphere, in such a way to preserve the first order focusing conditions of the analyzer. An energy resolution of approximately 30-35meV, 40-45 meV and 60-65 meV at 5 eV, 10 eV and 15 eV pass energies, respectively, is achieved in the entire kinetic energy range investigated.

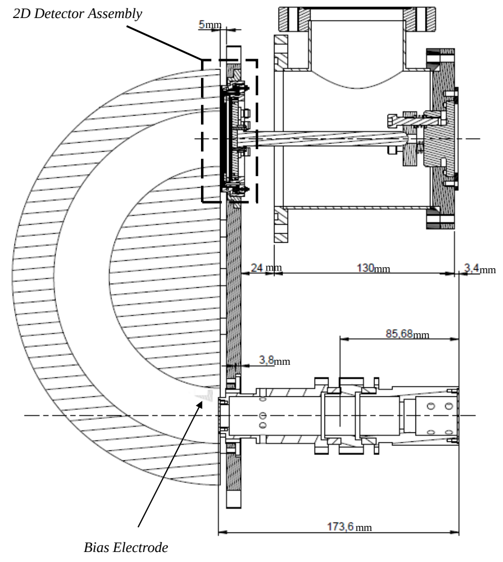

Figure S1: The cross-sectional view of the hemispherical analyzer housing the new 2D detector assembly. The newly developed asymmetric fringing field corrector is also shown (bias electrode).

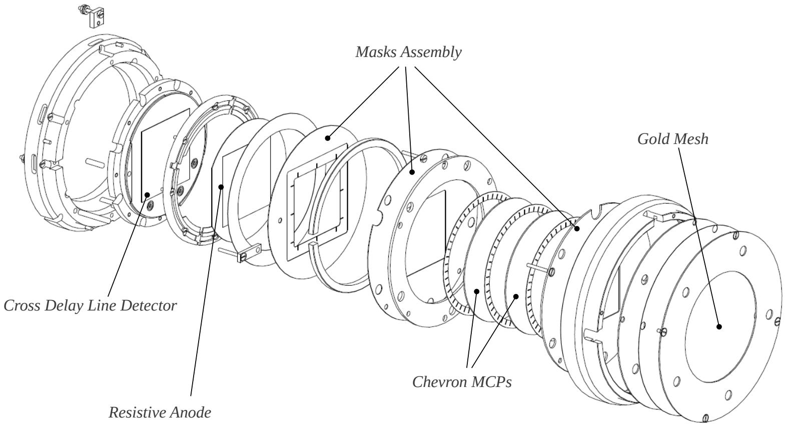

Figure S2: Exploded view of the two-dimensional cross delay line detector assembly mounted on the ARPES-TPES end station.

2 Outer valence PE spectra of OsO_4

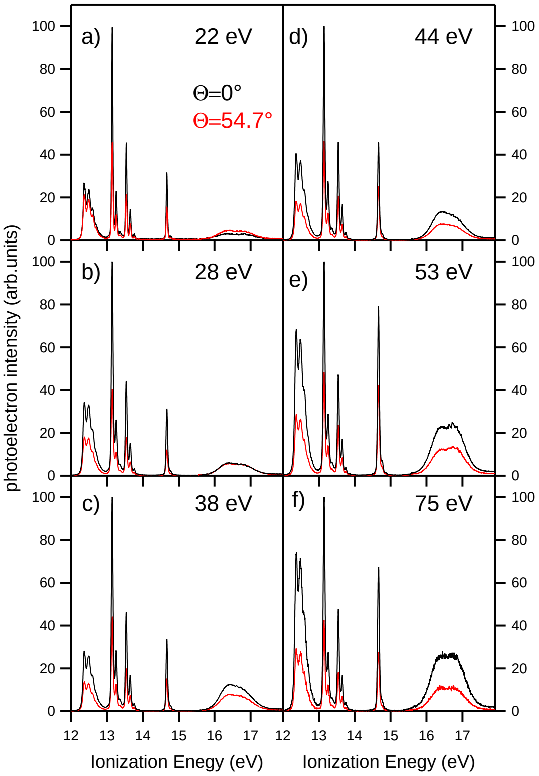

Figure S3: Outer valence PE spectra of OsO_4 (bands A-E) recorded at selected photon energies and at $\theta = 0°$ (black) and $\theta = 54.7°$ (red), which have been used to derive the β asymmetry parameters.

3 Valence ionization energetics of RuO_4, KS and TDDFT total photoabsorption, and photoionization σ and β profiles for selected valence and inner-valence MO ionizations of OsO_4 and RuO_4

The total photoionization cross section of the complexes, from threshold up to 150 eV photon energy, calculated at the KS and TDDFT levels of theory is reported in the upper and lower panels of Figure S4 for OsO_4 and RuO_4, respectively. For both complexes, the strong modulations observed at the TDDFT levels are due to the decay of singly excited states in the continuum (autoionizations), a manifestation of interchannel coupling effects, that cannot be described at the simpler KS level. The total photoionization cross section profiles are dominated by several well defined resonances at about 50 eV, which are due to the decay of the $np \longrightarrow nd$ giant resonance. As discussed in the text, this resonance preferentially decays in the 1e and $2t_2$ continua, in agreement with a previous work.[7] At higher photon energies, above 120 eV, the KS- and TDDFT-predicted cross sections are instead superimposed on the scale of Figure S4.

Table S1: Comparison between ZORA LB94/TZP MO energies and $\triangle$SCF IEs of RuO_4 from this work, and the experimental IEs taken from ref.[8] All energies are in eV. AO contributions to the MOs of RuO_4 calculated at the LB94/TZP level are also included.

Ion state (MO)	ΔSCF	$-\varepsilon_{KS}^{LB94}$	exp.[8]	AO character (LB94)
4U′ ($1t_1$)	12.51	15.07	12.15	O_{2p}
2E′ ($1t_1$)	12.70			
3U′ ($3t_2$)	13.30	15.83	12.92	96% O_{2p}/3% $Ru_{np} (n = 5, 6)$
2E″ ($3t_2$)	13.41		13.01	
1E′ ($2a_1$)	14.42	16.71	13.93	91% O_{2p}/4% O_{2s}/4% $Ru_{n's} (n' = 5, 7)$
1E″ ($2t_2$)	16.96	19.36	16.1	43% O_{2p}/8% O_{2s}/47% Ru_{4d}
2U′ ($2t_2$)	17.06		16.1	
1U′ (1e)	17.24	19.40	16.1	61% Ru_{4d}/40% O_{2p}

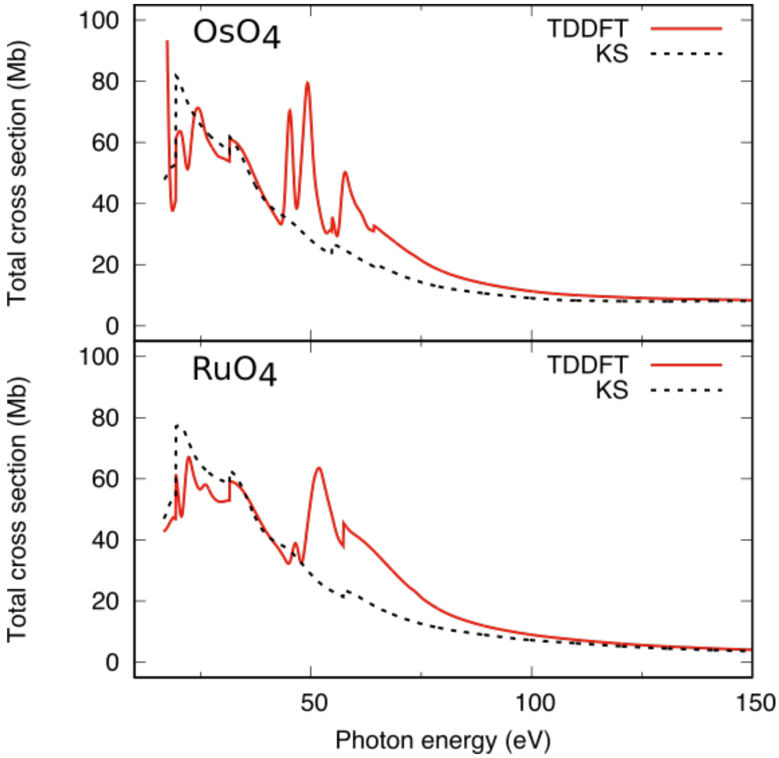

Figure S4: Total photoionization cross section of OsO_4 (upper panel) and RuO_4 (lower panel).

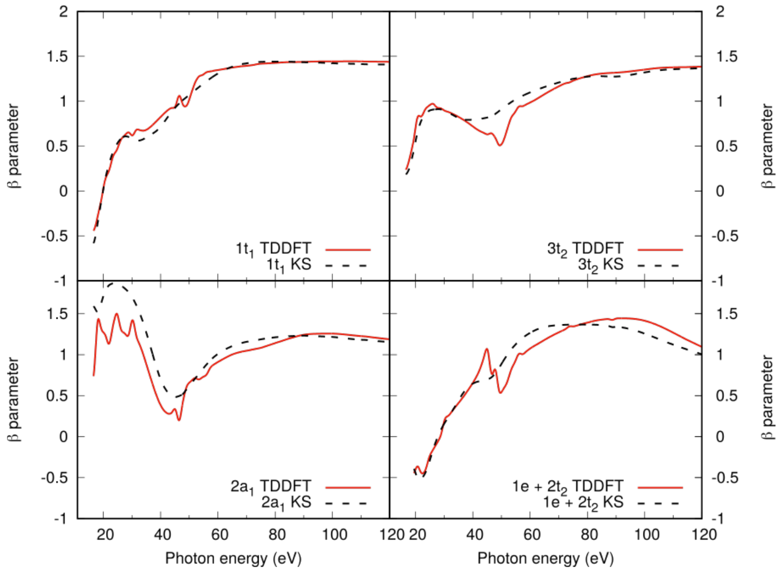

Figure S5: KS and TDDFT asymmetry parameter profiles for the outer valence ionizations of RuO_4.

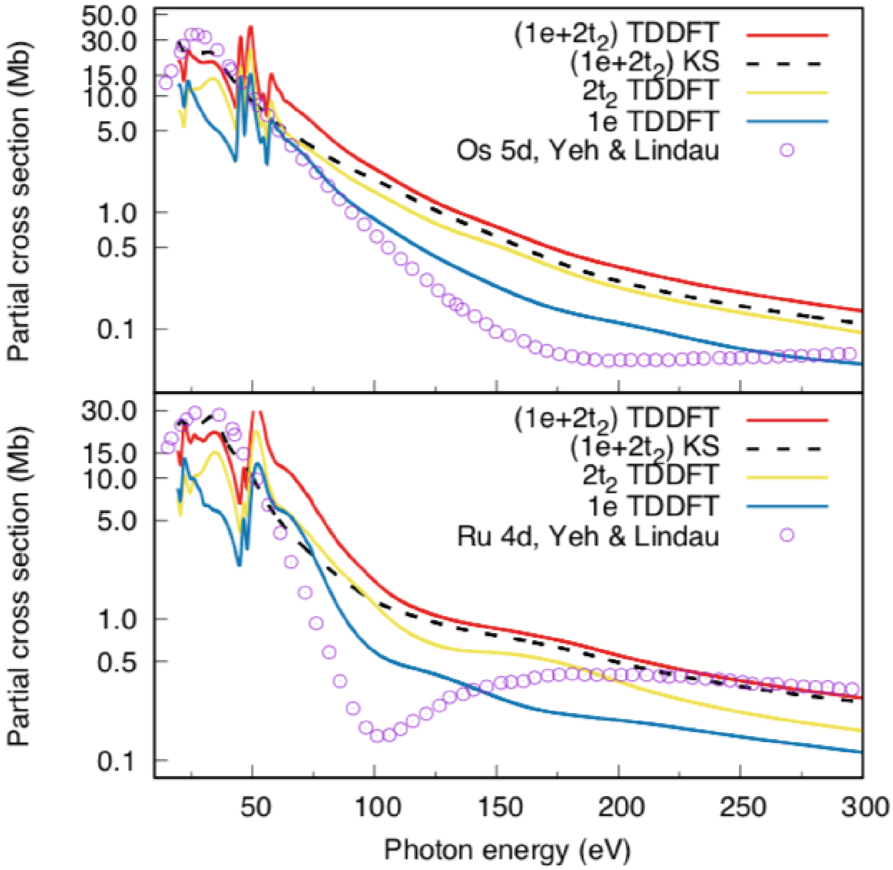

Figure S6: TDDFT partial cross section for the $(1e)^{-1}$ and $(2t_2)^{-1}$ionizations. Their sum, 1e + $2t_2$, is also shown, as calculated at KS and TDDFT levels, together with the d cross section of the metal atom calculated by Yeh and Lindau.[9] Upper panel: OsO_4; lower panel: RuO_4.

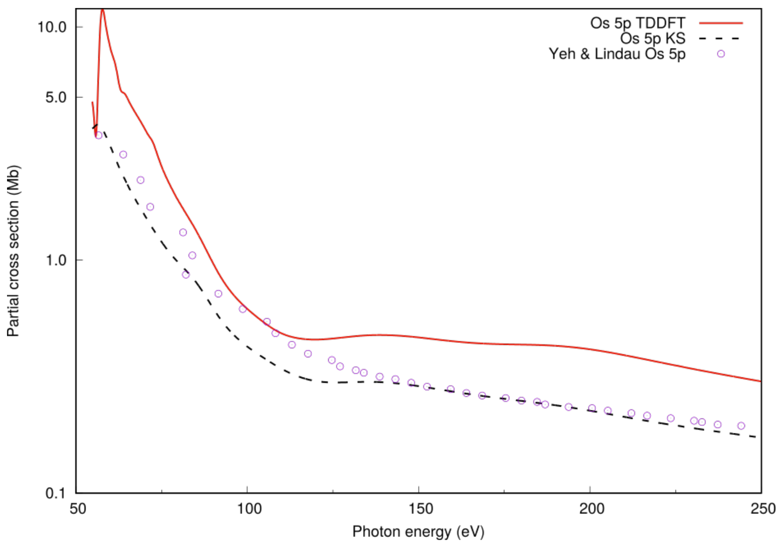

Figure S7: KS and TDDFT partial cross section for the Os $(5p)^{-1}$ ionization of OsO_4. Also reported for comparison is the atomic Os $5p$ theoretical estimate of Yeh and Lindau from ref.[9]

4 Ionization dynamics of selected ligand orbitals and metal semicore states

The partial cross section profiles for the O $2s$ inner-valence and metal semicore orbital ionizations in OsO_4 are presented in Figure S8, while the corresponding asymmetry parameter profiles are shown in Figure S9. Concerning the O $2s$ orbital ionizations, the KS results suggest the presence of a shape resonance at about 45 eV photon energy, which is followed by a rapidly decreasing partial cross section toward higher photon energies. The presence of this resonance in the TDDFT profile is obscured by the superposition of autoionization resonances converging to the Os $(5p)^{-1}$ ionization limit. Above approximately 150 eV photon energy, the KS and TDDFT profiles are essentially the same. The behavior of the Os $5p$ ionization cross section is quite interesting since it reveals at approximately 120 eV the presence of a Cooper minimum, which is, however, less pronounced in the KS profile. The ionization cross section of the Os $4f$ orbitals displays a delayed onset: its magnitude is small at 70 eV, right above threshold, and increases for higher excitation energies but with oscillations superimposed on a rising background. The ionization probability of

the Os $5s$ orbital is, instead, characterized by much smaller values compared to the other orbital ionizations, with broad maxima at about 120 eV and 200 eV photon energy. It is interesting to observe that interchannel coupling effects, which are responsible for the difference of the TDDFT values with respect to the KS ones, are rather ubiquitous: their importance in the near-threshold region is quite manifest in the O $2s$ and Os $5p$ ionizations as a large increase of the TDDFT curve relative to the KS one, while for the Os $4f$ and $5s$ ionizations, they affect the dynamics of the process over the whole photon energy region investigated.

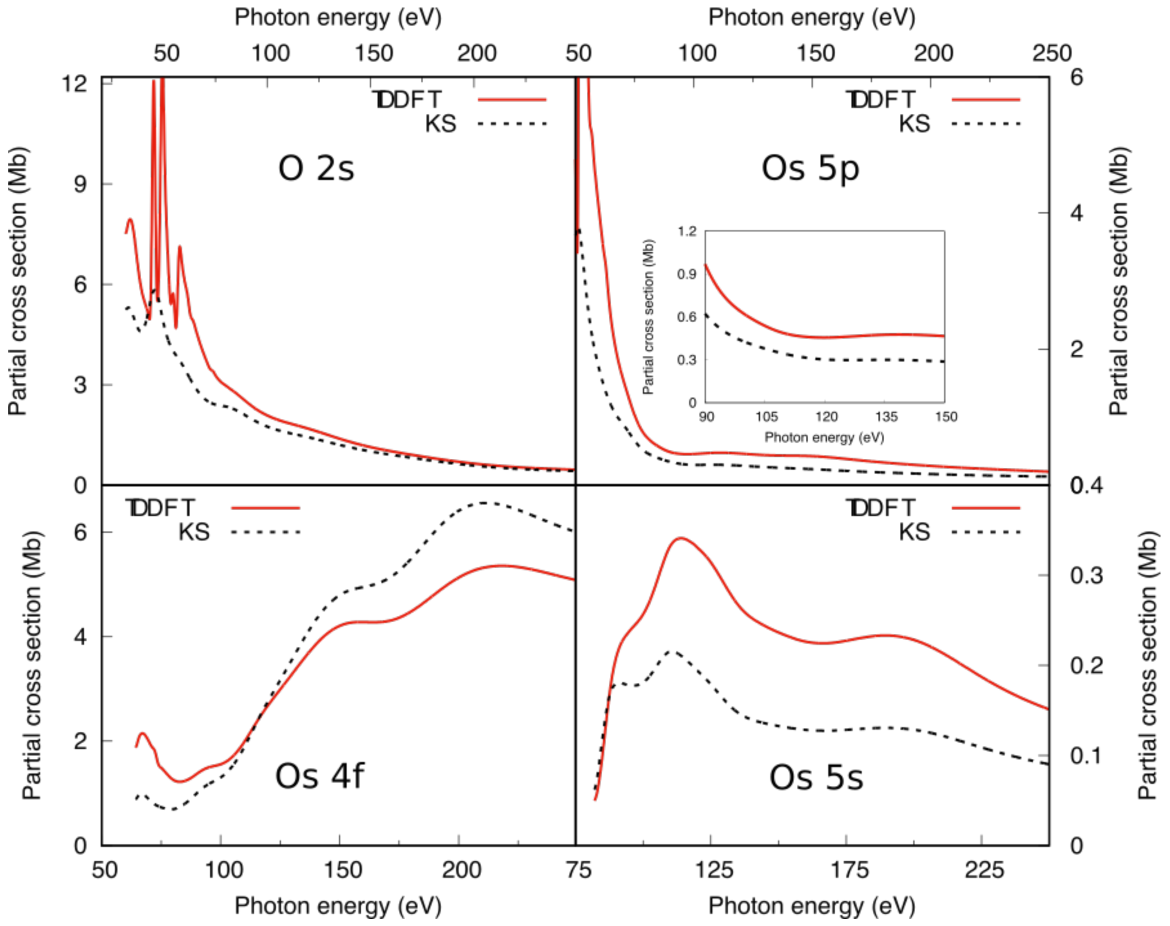

Figure S8: KS and TDDFT partial cross section profiles for selected MOs of OsO_4.

The KS and TDDFT calculated asymmetry parameters for ionizations of selected MOs of OsO_4 are reported in Figure S9 as a function of photon energy. The KS and TDDFT curves of the same ionization are very close to each other. This means that, on average, there is low sensitivity to

interchannel coupling effects. The only exception is represented by the Os $5p$ ionization where, although the curves show the same qualitative behaviour, the KS profile significantly underestimates the TDDFT predicted value, except for a narrow energy window just above threshold. Furthermore, while the β profiles for the O $2s$ and Os $5s$ ionizations are qualitatively similar, with a significant dip approximately 25 eV above threshold, the asymmetry parameter profile for the Os $4f$ orbitals starts with negative values close to threshold, then rises steeply to a maximum at about 80 eV photon energy, followed by a shallow minimum above 150 eV. A similar structure is observed for Os $5p$, although with a deeper minimum followed by a steeper increase. These features, characterizing the asymmetry parameter curves of the molecular photoionizations of OsO_4, are ascribed to the strong molecular field perturbation of the photoelectron continuum wave functions, because the initial core orbital, from which the electron is removed, is expected to be only little modified by the chemical environment. While the marked difference between the calculated molecular and atomic photoelectron asymmetry parameter curves is verified by comparing the results of the present work to those by Yeh and Lindau,[9] to our knowledge no experimental data have been published for the ionizations shown in Figure S9 to allow such a comparison.

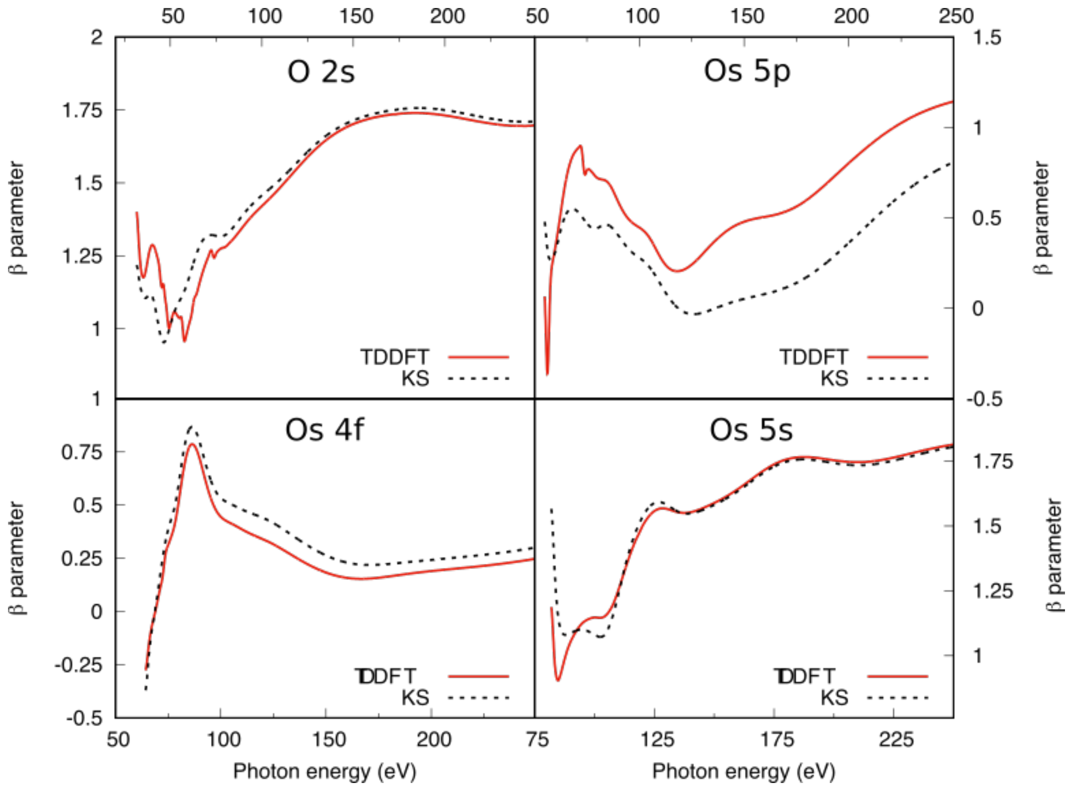

Figure S9: KS and TDDFT asymmetry parameter profiles for selected MOs of OsO_4.

Finally, partial cross section and asymmetry parameter profiles for the O$1s$ ionizations of OsO_4 are given in Figure S10. Apparent are the different magnitudes of the partial cross section for the $(5a_1)^{-1}$ and $(4t_2)^{-1}$ ionizations, as well as the presence of two resonant features (shape resonances), in the $(4t_2)^{-1}$ channel, around 15 eV and 50 eV photoelectron kinetic energy, while weak minima correspondingly appear in the $5a_1^{-1}$ channel. Since the $4t_2^{-1}$ cross section is much larger, and the two cannot be resolved, the summed profile is very close to the former. The high energy behaviour of the asymmetry parameters (lower panel of Figure S10) displays an out of phase oscillation for the channel resolved contributions, which is a manifestation of photoelectron interference effects.[10] The partial cross section and asymmetry parameter profiles for the O $2s$, O $1s$, Ru $4p$ and Ru $4s$ orbital ionizations have also been calculated. However, since their discussion mimics the one of the OsO_4 complex, we only collect the results below.

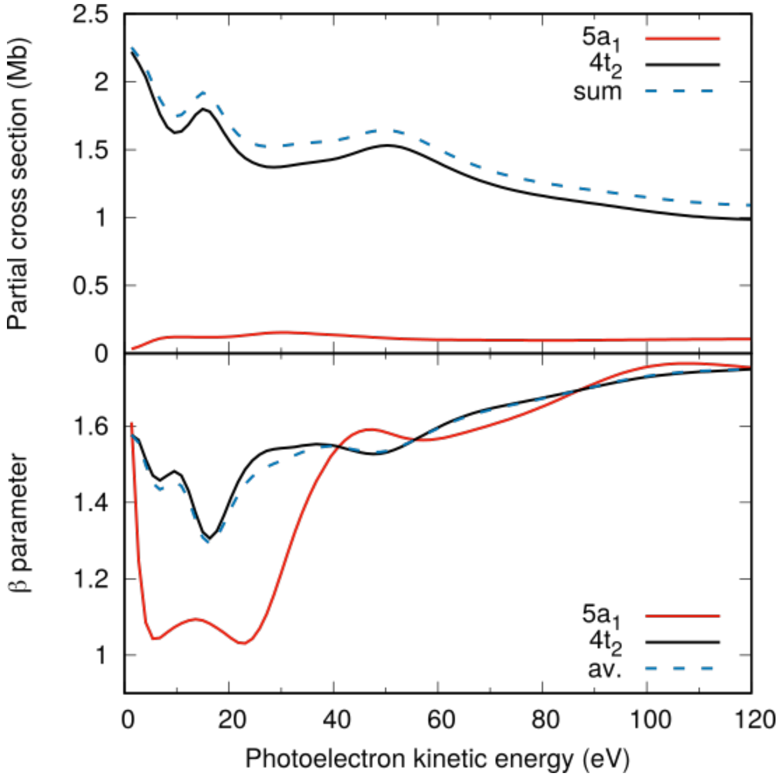

Figure S10: TDDFT partial cross section (upper panel) and asymmetry parameter (lower panel) profiles for the $(5a_1)^{-1}$ and $(4t_2)^{-1}$ MOs, corresponding to the O 1s ionization of OsO_4. The summed partial cross section and the average asymmetry parameter profile are also reported.

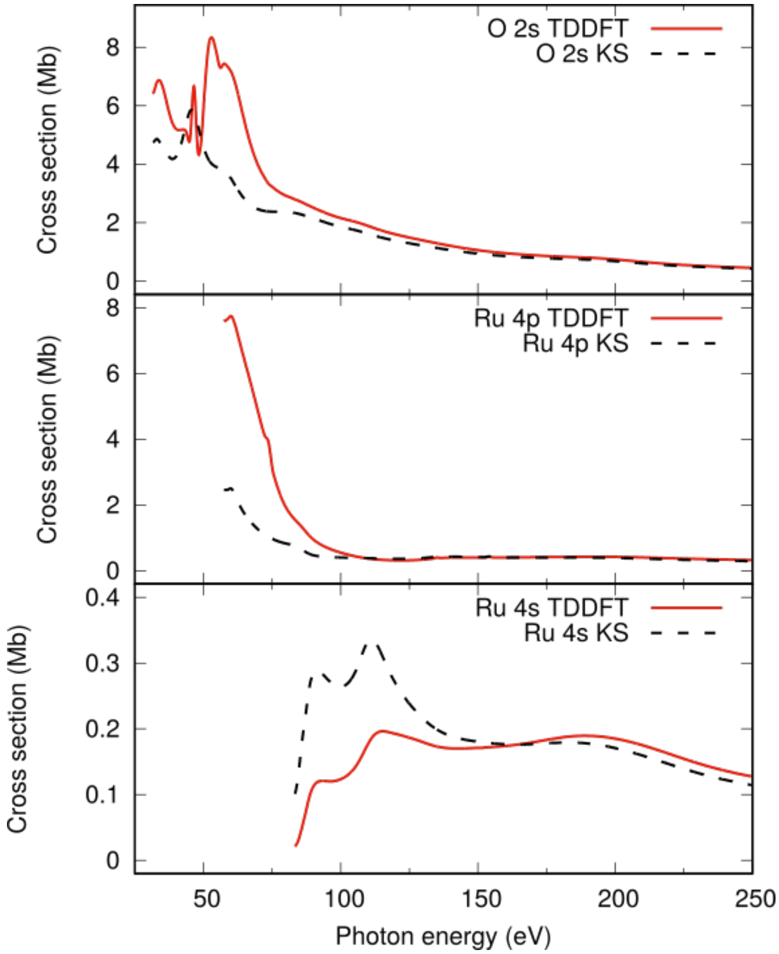

Figure S11: KS and TDDFT partial cross section profiles for selected MOs of RuO_4.

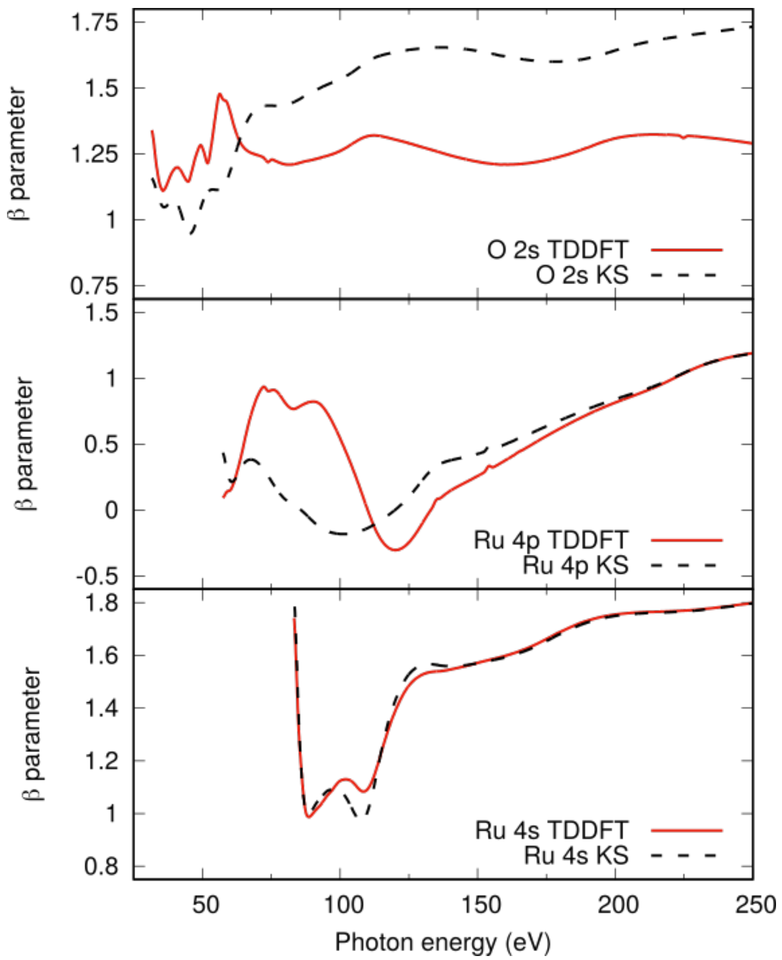

Figure S12: KS and TDDFT asymmetry parameter profiles for selected MOs of RuO_4.

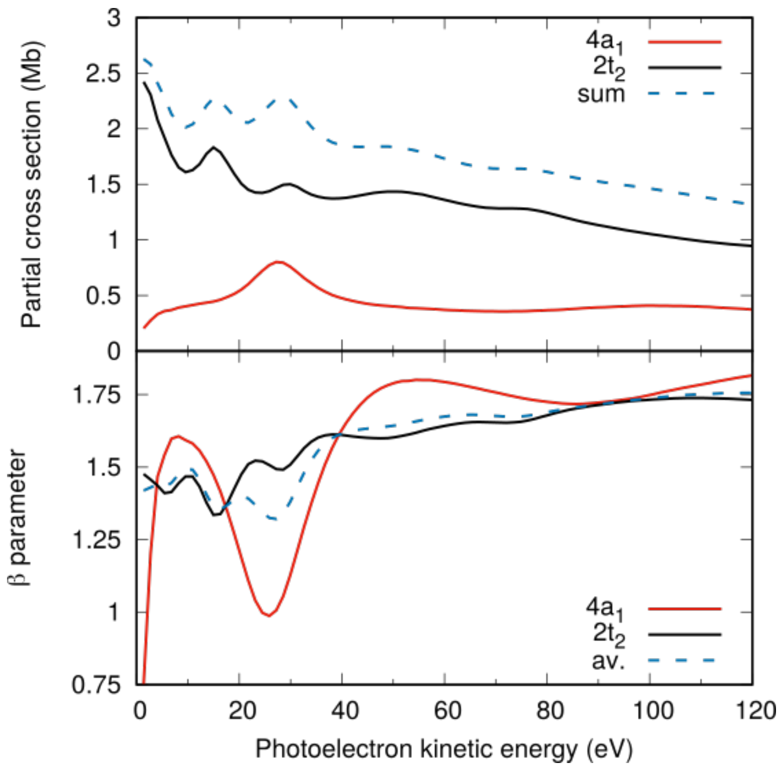

Figure S13: TDDFT partial cross section (upper panel) and asymmetry parameter (lower panel) profiles for the $(4a_1)^{-1}$ and $(2t_2)^{-1}$ MOs, corresponding to the O 1s ionization of RuO_4. The summed partial cross section and the average asymmetry parameter profile are also reported.

References

(1) to be published.

(2) The 2D cross delay line detector, as well as the whole data acquisition hardware and software development has been performed within a joint collaboration between the IOM-CNR Institution, the Elettra-Sincrotrone Trieste S.C.p.A., and the University of Rome ”La Sapienza”, Italy.

(3) Lampton, M.; Siegmund, O.; Raffanti, R. Delay line anodes for microchannelplate spectrometers. *Rev. Sci. Instrum.* **1987**, *58*, 2298–2305.

(4) Lampton, M.; Marckwordt, M. Stacked orthogonal serpentine delay lines with vias for two-dimensional microchannel plate readout. *Rev. Sci. Instrum.* **2000**, *71*, 4611–4619.

(5) Torelli, P.; Sacchi, M.; Cautero, G.; Cautero, M.; Krastanov, B.; Lacovig, P.; Pittana, P.; Sergo, R.; Tommasini, R.; Fondacaro, A. et al. Experimental setup for high energy photoemission using synchrotron radiation. *Rev. Sci. Instrum.* **2005**, *76*, 023909.

(6) Cautero, G.; Sergo, R.; Stebel, L.; Lacovig, P.; Pittana, P.; Predonzani, M.; Carrato, S. A two-dimensional detector for pump-and-probe and time resolved experiments. *Nucl. Instr. and Meth. A* **2008**, *595*, 447–459.

(7) Green, J. C.; Guest, M. F.; Hillier, I. H.; Jarrett-Sprague, S. A.; Kaltsoyannis, N.; MacDonald, M. A.; Sze, K. H. Variable photon energy photoelectron spectroscopy of osmium tetroxide and pseudopotential calculations of the valence ionization energies of OsO4 and ruthenium tetroxide. *Inorganic Chemistry* **1992**, *31*, 1588–1594.

(8) Burroughs, P.; Evans, S.; Hamnett, A.; Orchard, A. F.; Richardson, N. V. He-I photoelectron spectra of some d0 transition metal compounds. *J. Chem. Soc., Faraday Trans. 2* **1974**, *70*, 1895–1911.

(9) Yeh, J.; Lindau, I. Atomic subshell photoionization cross sections and asymmetry parameters: $1 \leq Z \leq 103$. *Atomic Data and Nuclear Data Tables* **1985**, *32*, 1 – 155.

(10) Decleva, P.; Toffoli, D.; Kushawaha, R. K.; MacDonald, M.; Piancastelli, M. N.; Simon, M.; Zuin, L. Interference effects in photoelectron asymmetry parameter (β) trends of C $2s^{-1}$ states of ethyne, ethene and ethane. *Journal of Physics B: Atomic, Molecular and Optical Physics* **2016**, *49*, 235102.
